# Towards whole plastome phylogeography: resolving small genetic distances among European *Arnica montana* L. with the PlastidPipeline

**DOI:** 10.1038/s41598-026-65517-1

**Published:** 2026-08-15

**Authors:** Siddharth J. Annaldasula, Manuel Vera, Adrián Casanova, Thomas Borsch, Katja Reichel

**Affiliations:** 1 Botanischer Garten und Botanisches Museum Berlin, Freie Universität Berlin, Königin-Luise-Straße 6-8, 14195 Berlin, Germany; 2 Facultade de Veterinaria, Instituto iTERRA, Departamento Zooloxía, Xenética e Antropoloxía Física, Universidade de Santiago de Compostela, Avenida Carballo Calero s/n, Lugo, 27002 Spain; 3 Institut für Biologie, Systematische Botanik und Pflanzengeographie, Freie Universität Berlin, Altensteinstraße 6, 14195 Berlin, Germany

**Keywords:** Arctic-alpine flora, Asteraceae, Clonal evolution, FAIR data, Holarctic floristic region, Phylogeny, Evolution, Genetics, Plant sciences

## Abstract

In phylogeographic studies, genetic distances between individuals are typically small: In the European endemic, declining medicinal plant *Arnica montana* L., range-wide biogeographic patterns so far remained obscure as sequence diversity in plastome marker regions is very low. While entire chloroplast genomes promise higher resolution, comparing them in great detail is a technical challenge, requiring high accuracy and repeatability in sequencing, assembly and annotation, automated across large sample sets. Using *A. montana* as our working example, we developed the PlastidPipeline, which consistently and repeatably provides structurally standardized and annotated, ready-to-analyze plastomes from short-read sequences. The PlastidPipeline combines well-tested standard software tools with own scripts, from data cleanup through plastome assembly, structural standardization, annotation, to quality control and raw read backmapping. Using publicly available read data of diverse provenance, we obtained plastid genomes for eight *A. montana* accessions across Europe, four further species of *Arnica* and three outgroup species. All *A. montana* plastomes formed a well-supported clade, split further into an Iberian and a Central/Northern European geographic group, which are, however, connected by patterns of potential heteroplasmy. Beyond demonstrating that pan-plastome phylogeography is both feasible and useful, we recommend the analysis of within-sample read variation, and improved standardization of newly reported plastomes.

**Supplementary Information:**

The online version contains supplementary material available at 10.1038/s41598-026-65517-1.

## Introduction

Chloroplast DNA (cpDNA) sequences have long served as an important source of information for phylogeographic studies in plants^1,2^. Early research focused on marker regions that could be readily amplified across a wide range of taxa^3,4^. More recently, whole chloroplast genomes (plastomes) have become available for an increasing number of species^5^ (> 8 000 species in GenBak as of February 2025), mostly through assembly from high-throughput short-read sequencing data^6,7^. Biodiversity sequencing initiatives such as the PhyloAlps, DNAmark and PhyloNorway have further accelerated this development^8–12^. Whole plastomes can be used either to identify informative marker regions^13–15^, or directly compared in a “pan-plastomic” framework^16–19^. The latter is especially useful when genetic differences are small, as expected in recent radiations^20^ or within-species phylogeographic studies. However, such analyses are highly sensitive to assembly and annotation errors and therefore require robust and standardized workflows.

Plastome reconstruction from short-read whole-genome shotgun (WGS) data poses several bioinformatic challenges. Raw datasets contain nuclear, mitochondrial and plastid reads, including sequences originating from intergenomic transfer such as nuclear plastid DNA insertions (NUPTs)^21^. In addition, alternative reads may arise from sequencing errors, PCR artefacts, DNA degradation, intra-individual polymorphism or heteroplasmy^22–27^. Plastid genome structure itself can also complicate assembly: beside the canonical quadripartite organization, with a large (LSC) and a small (SSC) single copy region separated by two inverted repeats (IR, or IRA/IRB for distinction), alternative structural configurations may coexist within individuals^28–31^, making some genomic regions difficult to assemble. Finally, repetitive regions approaching or exceeding read length can create ambiguities that are difficult to resolve using short-read data alone^32^.

To address these challenges, numerous pipelines for *de novo* plastome assembly have been developed^33–35^. Nevertheless, their performance and reliability differ considerably^36,37^. Consequently, the “linearized” plastome sequences deposited in public databases may not just flip between structural variants (Supplementary Figs. S1.1–3), but also contain assembly artefacts, structural inconsistences or annotation errors. Particularly when comparing closely related taxa, such issues can generate apparent genetic differences that are technical rather than biological. Re-assembly of publicly available raw sequence data using standardized workflows can therefore improve data quality and comparability across studies.

Although plastomes are often treated as a single non-recombining linkage group^38^ (excepting gene transfer^21,39^, levels of variation differ markedly among taxa and genomic regions^4,13^, raising questions about marker representativeness. In some groups, commonly used plastid markers provide insufficient resolution for species identification or phylogeographic inference^2,15^, yet harbour substantial variation elsewhere^28,40^. Intraspecific plastid diversity is often sufficient for phylogeographic studies^4^, but exceptions exist where even extensive screening of classic plastid markers yields only (almost) uniform sequences. One such example is *Arnica montana* L^41–45^.

The genus *Arnica* (Asteraceae) has 29 accepted species^41^ and a circumboreal distribution, with centers of diversity in western North America^46^. Although hybridization and polyploidy occur in some North American *Arnica* lineages^47,48^, *A. montana* is diploid and geographically isolated from all other *Arnica* species. No hybrids are known even with its geographically closest congener, *A. angustifolia*^49,50^. In the only plastid marker-based phylogeny of the genus, two *A. montana* samples from Sweden and France appeared as polyphyletic, although with very low support and poor overall resolution^41^.

*Arnica montana* ranges from the western Iberian Peninsula to western Ukraine and from northern Italy to central Norway, occupying naturally open habitats in mountains and ecologically similar lowland habitats^51,52^. Populations have declined strongly during the last century owing to land-use change, climate change and harvesting for medicinal purposes^44,50–52^. From the North-West of the Iberian Peninsula (Galicia and parts of Portugal), a subspecies *A. montana* subsp. *atlantica* has been described^53^, but later questioned since its morphological basis proved unreliable^54^. As the distribution of the subspecies stated in the protologue roughly corresponds to the geographic distribution of two plastid haplotypes, based on *rps16* intron and *pafII-cemA* (= *ycf4-cemA*) spacer sequences^42,55,56^, the taxon was upheld. Looking at nuclear microsatellite length polymorphism, another study found strong differentiation between Galician and Central European *A. montana* populations^57^. As the current distribution area of *A. montana* was strongly affected by glaciation during the Quaternary climate oscillations, these differences have been interpreted as the result of post-glacial re-colonisation from one (Galicia^42^) or more (Alps, Northern Europe^44,58^) glacial refugia. In general, the evolutionary relationships between *A. montana* populations in disjunct parts of its range, as well as between *A. montana* and other *Arnica* species, are currently not well known.

**Fig. 1:**
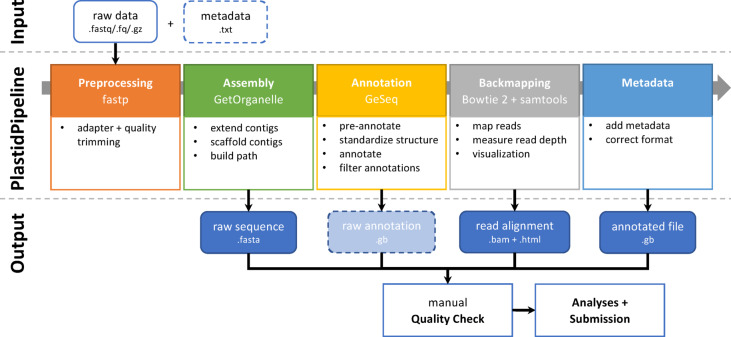
Schematic overview of the PlastidPipeline. Input, main output, subprocesses and main third-party tools used are specified for each step. More details are given in the Methods, and in the pipeline’s online documentation (https://github.com/sannalda/plastidpipeline).

To answer phylogeographic questions in cases such as *A. montana*, and improve the quality and reproducibility of plastome analyses more generally, we developed the PlastidPipeline (Fig. 1). This bioinformatic pipeline takes demultiplexed short-read data as input, performs quality filtering, plastome assembly, structural standardization, functional annotation and the generation of quality control files, i.e. assembly and annotation statistics, raw read backmapping files and zoomable read depth plots per sample. Its main output are daft annotated plastome files in a linearized format, which are directly ready for visual verification in a genome viewer, alignment and further analyses. Beyond integrating established tools such as GetOrganelle^59^ and GeSeq^60^ into a batch-mode snakemake^61^ pipeline, the PlastidPipeline contains convenience functions e.g. to automatically add basic metadata to the plastome files, truly standardize the sequence and direction of the four plastome parts and their annotations (thus correcting bugs in both GetOrganelle and GeSeq, Supplementary Figs. S1.1–3), and check which plastome genes may be missing from a standard list. Compared with the plastaumatic pipeline^62^, it uses GetOrganelle instead of NOVOPlasty based on more reproducible assemblies^36^, employs GeSeq for annotation, and eases quality-control procedures through systematic backmapping and visualization steps. The pipeline and documentation are available on GitHub (link), with all code released under the MIT licence^63^.

Using all publicly available and some new short-read datasets for *A. montana* and related species, which differ in size, original project and physical source material, we seek to answer three questions:

1. How does the PlastidPipeline facilitate the production and analysis of plastomes, and which potential issues remain?
2. Using all currently available data, what can plastomes tell us about the phylogenetic origin and phylogeography of *A. montana*?
3. Which advantages does whole plastome-based phylogeography have?

**Fig. 2:**
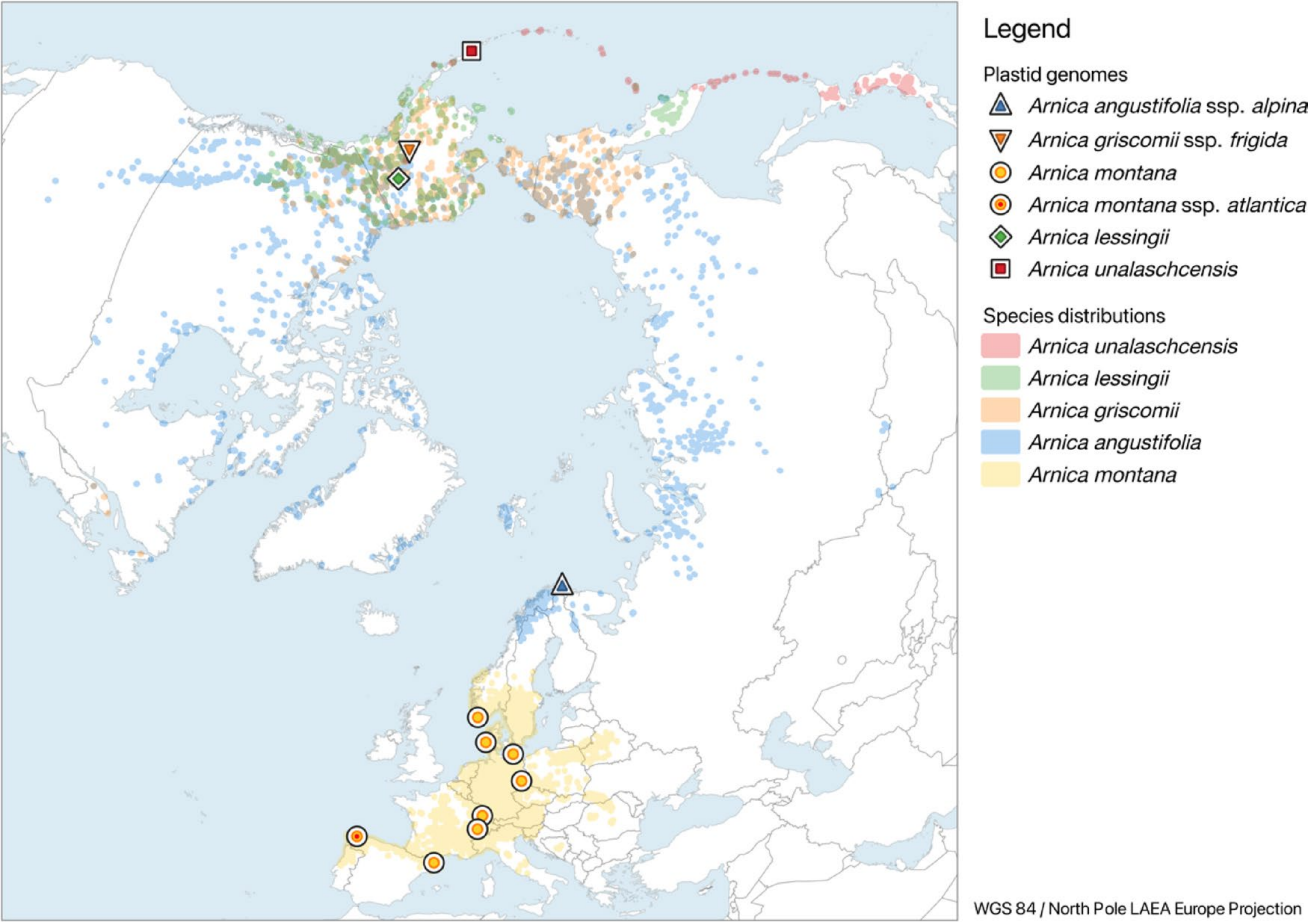
Distribution map and sample origin of *Arnica* species included in this study. Distribution areas of putative subspecies of *A. angustifolia* Vahl and *A. frigida* C.A. Mey. ex Iljin are not distinguished. Dots appearing grey result from overprinting of different species’ occurrences. Based on data from gbif.org (see text).

To study these, we compared plastome diversity across three hierarchical levels: among *Arnica* species, among *A. montana* accessions, and within individual *A. montana* read datasets. Given the limited sampling, coming from one accession each of three outgroup species and four Arctic *Arnica* species, plus eight *A. montana* accessions collected throughout Europe (Fig. 2), our results should be regarded as a proof of concept and a source of hypotheses for future studies rather than as definitive conclusions regarding the evolutionary history of *A. montana*.

## Results

### Statistic analysis

For each of the 23 datasets obtained from 15 samples (Table 1), including six technical replicates (runs from the same DNA library) for one sample and downsampled versions^36^ of the two largest datasets, we assembled and annotated a complete circular plastid genome sequence. Assembly parameters and statistics are given in Table 2 and Supplementary Table S2.1. In the quality filtering step, the datasets from PCR-free library preparation did not contain a lower percentage of duplicate reads; we therefore did not specifically filter these reads out. After the initial batch run for all datasets with default settings, the number of extension rounds R and word size w had to be manually adjusted for several cases, which were subsequently re-run in sub-batches to get entire plastomes for all samples.

**Table 1:** Overview of specimens included in this study. Sample ID: a four-letter species code and the two-letter International Standards Organization (ISO) code for the country, or state/province of origin if multiple samples per country. Group: *post hoc* geographic group within *A. montana* (see Results), CNE Central/Northern Europe, IB Iberian Peninsula. cult. BG cultivated in Botanic Garden, * no coordinates available, IDs for herbarium vouchers preceded by Herbarium Code and a colon.

Sample ID	Species	Lat	Long	Group	Locality	Further IDs	Project	Reference
Amon_AG	*Arnica montana*	58.21	6.57	CNE	Norway, Agder, Rasvåg on Hidra Island	TROM: TROM_V_59412	PhyloNorway	^8^
Amon_DK	*Arnica montana*	55.65	8.17	CNE	Denmark, Syddanmark, Børsmose	DM447	DNAmark	^10^
Amon_MV	*Arnica montana*	54.39	12.70	CNE	Germany, Mecklenburg-Western Pommerania, Barth	AM09_09	BO Berlin	^44^/ This study
Amon_BB	*Arnica montana*	51.51	13.77	CNE	Germany, Brandenburg, Lauchhammer / cult. BG Berlin	B:B 10 1154693, AM0057	ArnicaSNP	This study
Amon_BW	*Arnica montana*	47.86	8.02	CNE	Germany, Baden-Württemberg, Feldberg	AM21_34	BO Berlin	^44^/ This study
Amon_CH	*Arnica montana*	46.39	7.42	CNE	Switzerland, Bern, Lenk	GR:GR003708, PHA000838	PhyloAlps	^12^
Amon_CT	*Arnica montana*	42.33	2.23	IB	Spain, Catalonia, Vilallonga de Ter / cult. BG Barcelona	BC:BC-997190	rDNA-LIFE	^64^
Amon_GL	*Arnica montana* subsp. *atlantica*	43.28	−7.86	IB	Spain, Galicia, Ponte Pedrido / cult. USC Lugo	AY5634	CONSERVARNICA	This study
Aang_FI	*Arnica angustifolia* subsp. *alpina*	70.62	29.81	–	Norway, Finnmark, Båtsfjord	TROM: TROM_V_962577	PhyloNorway	^8^
Agri_AK	*Arnica griscomii* subsp. *frigida*	63.05	-151.00	–	USA, Alaska, Denali National Park	TROM: TROM_V_300499	PhyloNorway	^8^
Ales_AK	*Arnica lessingii*	65.36	−146.41	–	USA, Alaska, White Mountains	TROM: TROM_V_300501	PhyloNorway	^8^
Auna_AK	*Arnica unalaschcensis*	54.16	-165.93	–	USA, Alaska, Akutan Island	TROM: TROM_V_300511	PhyloNorway	^8^
Ecan_CT	*Eupatorium cannabinum*	42.37	2.30	–	Spain, Catalonia, Setcases	BC:BC-997195	rDNA-LIFE	^64^
Gtul_MX	*Guardiola tulocarpus*	–	–	–	Mexico*	TEX:Panero 3062	Asteraceae Phylo-transcriptomics	^65^
Vcar_CA	*Venegasia carpesioides*	–	–	–	USA, California, Santa Barbara area*	JEPS:Baldwin 893	Asteraceae Phylo-transcriptomics	^65^

**Table 2:** Overview of read and assembly statistics for all datasets in this study. Reads: read length, Pairs: no. of raw read pairs, Filtered: no. of read pairs after quality filtering, Dupl.: percentage of reads with identical sequence in the data, R: GetOrganelle extension rounds for assembly, w: GetOrganelle wordsize for assembly, Mem.: memory needed for assembly, Time: wall-clock time for assembly in minutes, Plastome: plastome length, LSC/SSC/IR: length of respective plastome part, Insert: mean insert size of assembled read pairs, Depth: mean and minimum values for read depth on the assembled plastome, Mapped: percentage of raw reads mapping to the assembled plastome.

Dataset	SRA run ID	Library prep	Reads [bp]	Pairs [M]	Filtered [M]	Dupl. [%]	R	w	Mem. [Gb]	Time [min]	Plastome [bp]	LSC [bp]	SSC [bp]	IR [bp]	Insert [bp]	Depth mean	Depth min	Mapped [%]
Amon_AG	ERR8268641	with PCR	100	3.37	3.32	0.46	30	66	3.2	18	152 089	83 843	18 304	24 971	256	68.7	21	3.09
Amon_DK	SRR12432532	with PCR	150	8.08	7.56	2.14	27	65	38.0	213	152 090	83 844	18 304	24 971	228	307.3	52	4.35
Amon_MV	ERR15302247	with PCR	150	22.86	22.27	2.95	30	85	60.55	177	152 091	83 845	18 304	24 971	449	2 338.0	1 421	10.31
Amon_BB	ERR15310125	PCR-free	150	675.51	648.56	4.68	30	112	14.97	61	152 070	83 843	18 279	24 971	356	68 016.9	29 153	10.17
Amon_BB-d	*downsampled*	PCR-free	150	36.17	35.11	0.24	30	90	9.83	124	152 070	83 849	18 279	24 971	327	127.9	68	0.36
Amon_BW	ERR15302248	with PCR	150	18.30	17.69	2.08	30	112	17.08	111	152 094	83 849	18 303	24 971	449	403.4	110	2.22
Amon_CH	ERR14008899	with PCR	150	5.35	5.35	0.18	11	85	8.28	8	152 087	83 842	18 303	24 971	330	193.8	78	5.56
Amon_CT	SRR17032105	with PCR	150	4.77	4.39	2.51	30	84	14.24	41	151 989	83 750	18 297	24 971	382	149.7	32	3.18
Amon_GL	*aggregate*	PCR-free	150	361.65	354.78	2.29	30	112	16.58	96	151 999	83 757	18 300	24 971	420	19 637.9	11 820	5.47
Amon_GL-d	*downsampled*	PCR-free	150	224.83	220.94	1.88	40	100	29.1	142	151 999	83 716	18 300	24 971	451	385.3	184	0.17
Amon_GL-71	ERR15340092	PCR-free	150	189.51	186.21	1.88	30	112	16.58	99	151 999	83 757	18 300	24 971	420	10 299.1	6 378	5.48
Amon_GL-72	ERR15340095	PCR-free	150	192.20	189.22	2.13	30	112	15.98	89	151 999	83 757	18 300	24 971	428	10 466.5	6 378	5.49
Amon_GL-73	ERR15340093	PCR-free	150	190.09	185.11	1.89	30	112	15.73	95	151 999	83 757	18 300	24 971	433	10 238.8	5 920	5.43
Amon_GL-74	ERR15340094	PCR-free	150	184.84	181.84	2.79	30	112	16.52	96	151 999	83 757	18 300	24 971	425	10 090.4	6 062	5.50
Amon_GL-Y1	ERR15337219	PCR-free	150	60.38	59.15	2.22	30	112	15.83	89	151 999	83 757	18 300	24 971	409	3 286.0	2 188	5.49
Amon_GL-Y2	ERR15336770	PCR-free	150	61.16	59.93	2.19	30	112	3.97	23	151 999	83 757	18 300	24 971	411	3 330.0	2 171	5.49
Aang_FI	ERR5554746	with PCR	100	3.83	3.75	1.21	24	66	3.47	14	152 052	83 809	18 301	24 971	289	177.3	80	7.01
Agri_AK	ERR5529436	with PCR	100	4.45	4.39	1.00	30	65	3.97	23	151 981	83 733	18 306	24 971	222	178.6	43	6.10
Ales_AK	ERR5529317	with PCR	100	4.22	4.18	0.40	30	65	5.97	21	152 056	83 840	18 274	24 971	196	83.7	17	3.01
Auna_AK	ERR5529299	with PCR	100	6.12	6.06	0.35	12	85	10.18	12	152 008	83 716	18 262	25 016	208	120.8	15	3.00
Ecan_CT	SRR17032099	with PCR	150	3.99	3.74	3.10	30	112	6.21	20	151 384	83 024	18 312	25 024	376	370.0	80	9.36
Gtul_MX	SRR12917849	with PCR	150	22.40	21.55	1.56	17	111	12.15	52	151 368	83 219	18 163	24 993	236	1 754.4	900	7.97
Vcar_CA	SRR12917857	with PCR	150	16.57	15.72	1.69	30	110	10.85	70	152 351	83 866	18 326	25 037	243	1 346.4	521	8.38

From the backmapping statistics, we determined the shares of plastome reads in the raw datasets (Table 2). Excluding the downsampled data (both < 1%), these had a median of 5.5% and ranged from 2.2% (Amon_BW) to 10.3% (Amon_MV). Both libraries were prepared in parallel from high-quality *A. montana* DNA in the same lab. Plastomes with mean read depths between 100 and 1 000 still showed considerable variation in their read depth minimum: in Auna_AK, mean read depth is well over 100, yet local read depth at one locus dropped as low as 15 (12.4%). With the exception of Vcar_CA and Amon_BB, samples with high mean read depths of > 1 000 also had more even read depth, indicated by minimum: mean read depth ratios from 0.5 to 0.7. The herbarium material-derived libraries (and downsampled datasets) mostly had low ratios and low minimum read depths, but were also generally smaller due to their original project’s sequencing strategies, thus confounding the effects of source material and sequencing effort. We did not find correlations between input file characteristics (read pairs, percentage of reads mapping to plastome) and computational effort (memory use, wall-clock time; Supplementary Table S2.1).

Comparing technically different plastome assemblies for the same biological sample, plastomes from the same DNA library (Amon_GL, technical replicates) were always identical. Whereas assemblies derived from original and downsampled datasets were also identical for Amon_BB, Amon_GL-d contained one insertion (10 bp, minisatellite) and one deletion (51 bp) compared to Amon_GL. The technical replicates and backmapping alignment files gave more support to the non-downsampled version, which was subsequently used. Multiple pipeline runs with the same parameters consistently produced the same assembled sequences for our data, yet slight parameter changes sometimes had an effect: for the Vcar_CA dataset, assemblies with different pre-set numbers of assembly rounds (R = [10, 15]) differed by 7 bp in the length of an A/T-homopolymer in the *atpE-ndhC* spacer. A comparison with the read depth graph produced by the PlastidPipeline, where the region in question shows a considerable drop, and with a previously published NOVOPlasty assembly^66^ of the same dataset, in which the polyA/T-stretch is only 31 bp, not 85–78 bp long, suggests that this polymorphism may be an assembly artefact. A previous assembly was also available for Gtul_MX, yet turned out identical to ours.

**Fig. 3:**
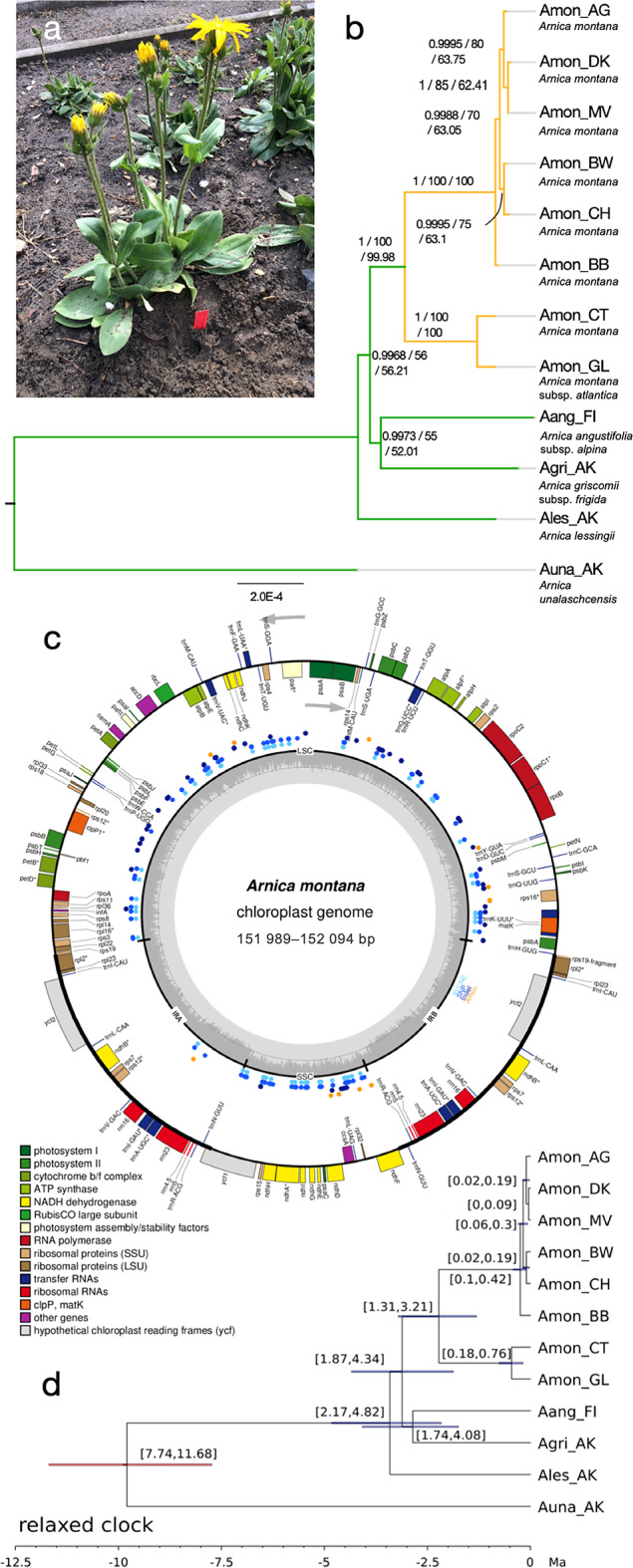
(**a**) *A. montana* habitus. (**b**) Phylogram of the Bayesian consensus tree for all *Arnica* plastomes in this study. Based on SNPs and 2-state-indels only, multi-state indels removed, *A. unalaschcensis* used as outgroup (see text). Tree topology is identical to the most strongly supported Parsimony and Maximum Likelihood trees. Branches labeled with posterior probability (MrBayes) and percentage support values (RAxML unpartitioned data / PAUP* parsimony) based on 10 000 bootstrap samples, *A. montana* clade highlighted in yellow. (**c**) Pan-plastome map of *A. montana*. Species-characteristic SNPs (yellow), intraspecific indels (dark blue) and SNPs (mid blue), and geographic region-specific SNPs (light blue) marked by dots. (**d**) Dated *Arnica* phylogeny, assuming a relaxed molecular clock. Maximum credibility chronogram obtained from BEAST, showing branch length proportional to time. Bars indicating 95% highest posterior densities for node age, red bar marking the estimate for the branching point used in secondary calibration with two alternative priors (red). Oldest and youngest age estimates in millions of years (Ma) based on^69^(time scale, full color), or based on^65^ (faded).

The assembled plastomes for the non-*Arnica* samples were less uniform in size than within the genus *Arnica*, where all except the Auna_AK sample had exactly the same IR length (Table 2). Within *A. montana* (Fig. 3a), the LSCs of the two Iberian samples (Amon_GL, Amon_CT) were both at least 85 bp shorter, while differences in SSC lengths were minimal. According to the annotation checkfiles, all *Arnica* plastomes contained the same 86 (80 unique) coding genes, 36 (29 unique) tRNA genes and eight (four unique) rRNA genes. They did not contain *chlB*,* chlL*,* chlN*,* psaM* and *trnP*-GGG, as expected for angiosperms^67,68^ but also lacked *trnR*-CCG, for which there is a functional paralog, and *trnE*-UUC, the only gene for glutamic acid transfer RNA. Non-canonical start codons were detected for *psbC*, *rps19* (both GTG) and *psbC*,* psbL* (both ACG). In Amon_CH and Gtul_MX, the *matK* gene had a premature stop codon, caused by a frameshift mutation due to a polyA/T length polymorphism and leading to a loss of 81 amino acids (16%) of the translated sequence. Looking at the backmapping read alignment files, variant reads included a full-length version of the poly-A/T-stretch at a frequency of 2.5% (5 reads) in Amon_CH, and 1.8% (29 reads) in Gtul_MX.

### Plastome variation in the genus *Arnica*

All *A. montana* plastomes we analysed formed a highly supported clade (Fig. 3b). Apart from the early divergence of Aleutian / NE Asian *A. unalaschcensis*, the relationships between the plastomes of European (*A. montana*) and the other Beringian (*A. griscomii*,* A. lessingii*) or circumpolar (*A. angustifolia*) *Arnica* species were not well supported (Fig. 3b, Supplementary Fig. S1.4 including non-*Arnica* plastomes). While Neighbor-Joining did not resolve them at all, all other phylogenetic inference methods depicted *A. lessingii* + (*A. montana* + (*A. griscomii* + *A. angustifolia*)) as the best-supported topology. Partitioning data into LSC, IR and SSC (Fig. 3c) for RAxML did not change this result. Tentative molecular clock analyses, using the range of median values for the split between *A. montana* and *A. chamissonis* (not included in our study) estimated from transcriptome data under two different primary calibration scenarios^65,69^ as a secondary calibration for the divergence time of *A. unalaschcensis*, put the stem age of *A. montana* at [1.16, 4.04] Ma (strict molecular clock, Supplementary Fig. S1.5) or [0.94, 4.75] Ma (relaxed molecular clock; Fig. 3d).

The IR has least polymorphism among plastomes of different *Arnica* species. Based on single nucleotide polymorphisms (SNPs) only, the plastome regions best distinguishing *A. montana* from all other *Arnica* species in this study were the *rps16* intron and *ndhF* gene, with two SNPs each (Fig. 3c, Supplementary Table S2.2). The *trnQ*-UUU gene, *trnY-rpoB* spacer, *trnG-trnR* spacer, *psbD* gene, *trnV*-UAC intron, *rbcL* gene, *trnI*-GAU intron, *ndhD* gene and *ccsA* gene each contained one SNP. This corresponds to two amino acid changes in the *rbcL* (Glu > Lys) and *ccsA* (His > Asn) protein sequences potentially characteristic for *A. montana*. Apart from the *rps16* intron, none of these regions were previously studied in *Arnica*.

**Fig. 4:**
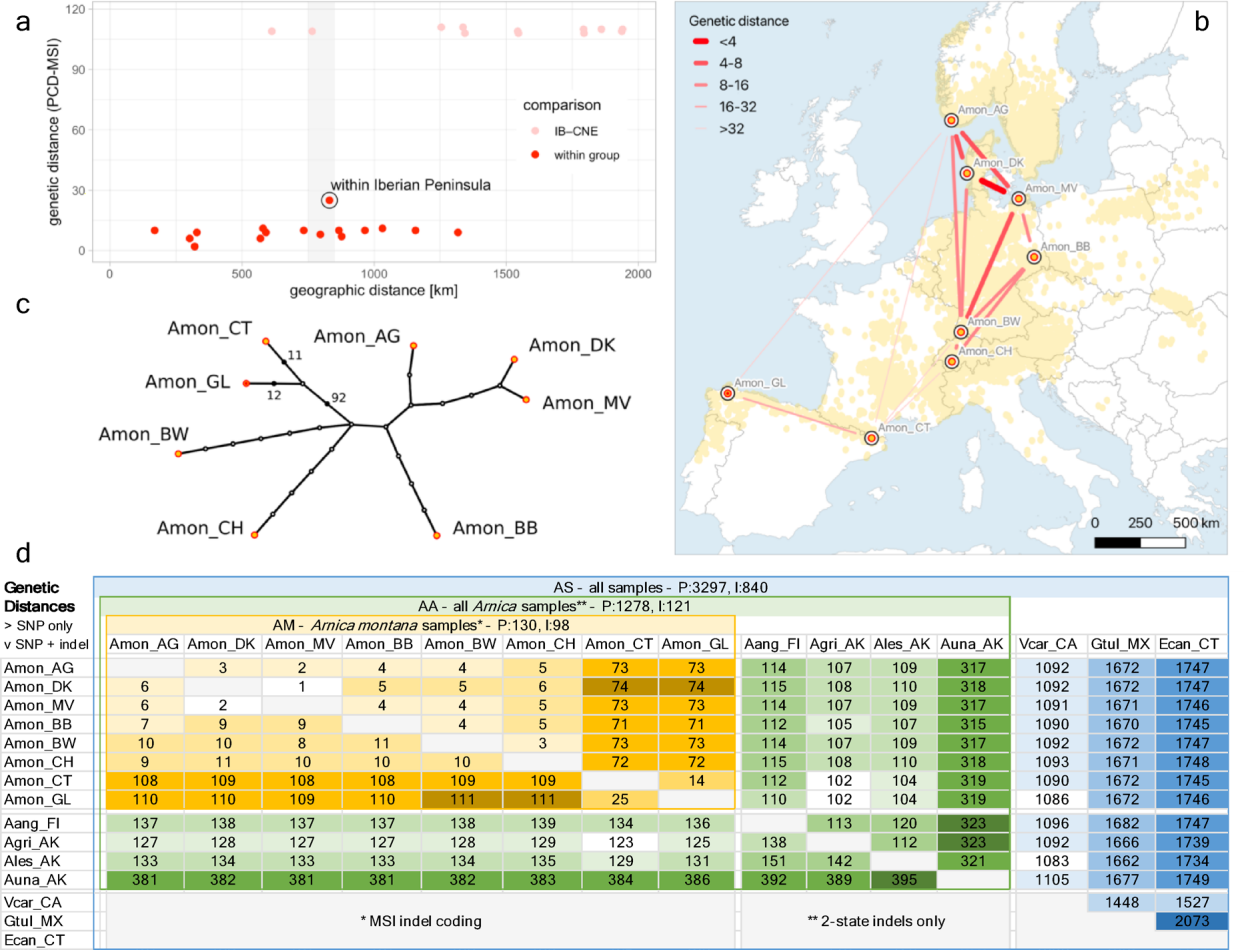
(**a**) Mantel plot of geographic and genetic distances between all *A. montana* plastomes/samples, based on the AM-MSI alignment (all *A. montana* plastomes with multistate indel coding, MSI). Distances within same *post hoc* geographic group, Iberian Peninsula (IB) or Central/Northern Europe (CNE) in red, between geographic groups in light red. Grey rectangle denotes geographic distance range discussed in the text. (**b**) Map and selected pairwise genetic distances between *A. montana* plastomes. Heavy/dark red lines correspond to low genetic distances. *A. montana* sampling sites and distribution area in yellow. (**c**) Parsimony network of *A. montana* plastomes based on the AM-MSI alignment. Yellow-red nodes observed, black nodes putative ancestral genotypes with one-step mutational distances. Distances above ten steps condensed and marked with number of collapsed nodes. (**d**) Heatmap of genetic distances between all studied plastomes. Pairwise character distance (PCD) based on SNPs only (above diagonal) or both SNPs and indels/inversions (below diagonal), colored by underlying alignment (AM – yellow, AA – green, AS – blue) from lowest (white) to highest (dark) values within each partial dataset. Indels removed (AS), only indels part of 2-state characters included (AA), or all indels coded as unordered multistate characters (AM). The total number of polymorphic sites (P) and parsimony-informative sites (I), also including gaps, are stated for each alignment.

### Plastome variation in *Arnica montana*

The plastomes of *A. montana* showed a clear divergence between Central/Northern European (CNE) and Iberian (IB) samples (Fig. 4a,b). We therefore included these two geographic groups as a *post hoc* classification in our analyses (Table 1). The resulting bimodal distribution of genetic distances causes a spuriously significant Mantel test (r_M_ = 0.687, *p* = 0.007): at a similar geographic distance of ca. 750–850 km, the pairwise genetic distance between CNE samples is smaller than between the two IB samples, and much smaller than between samples belonging to different groups (Fig. 4a). Polymorphism at the two plastid regions, *pafII-cemA* and the *rps16* intron, previously used to distinguish *A. montana* subsp. *atlantica* in Galicia and *A. montana* subsp. *montana* in the Eastern Pyrenees^56^, France and Belgium^45^, did not follow our expectations: none of the samples in this study showed polymorphism in the *rps16* intron, yet Amon_CT had the same, divergent genotype as Amon_GL at *pafII-cemA*, corresponding to the cHap1 (both IB) and cHap3 (CNE) combined haplotypes^42^. A comparison with previously published sequences of additional plastid markers (Supplementary Table S2.8) from other *A. montana* individuals, including one from an unspecified location in France^41^, yielded no further information.

Among the CNE samples, the Mantel test was not significant (r_M_ = 0.373, *p* = 0.069), but plotting pairwise distances on a map showed gradually less genetic distance between samples towards the North, and less total polymorphism than between the two Iberian samples (Fig. 4b). This corresponds with the parsimony network (Fig. 4c), and in part with the phylogenetic tree (Fig. 3b), which is, however, based on an alignment simplified by removing complex (multi-state) indels and thus less informative about relationships within *A. montana*. Based on just 21 polymorphic (six parsimony-informative) sites, genetic distances between CNE samples ranged from two to eleven (Fig. 4d), with the lowest genetic distance (1 Indel + 1 SNP) between the North German (Amon_MV) and Danish (Amon_DK) sample, collected at a direct geographic distance of ca. 320 km.

With two exceptions in the *rrn16* and *trnA*-UGC genes, polymorphism among the *A. montana* plastomes was concentrated in the single copy regions (Supplementary Tables S2.3, S2.6). Of 67 phylogenetically informative SNP loci across all *A. montana* samples, 62 clearly supported the IB/CNE split, with either group in turn possessing the (ancestral) allele also found in other *Arnica* species (IB: 33, CNE: 25 SNPs). The regions containing most SNPs across all studied *A. montana* plastomes were the *matK* gene (3 SNPs) and 5’ adjacent part of the *trnK* intron (1), *trnS*-GCU-*trnC*-GCA spacer (3), *rpoC2* gene (3), *ycf1* gene (8), *ndhA* intron (4) and adjacent *ndhA* exon 2 (1), *ndhI-ndhG* spacer (4), *rpl32-trnL*-UAG spacer (3), *rpl32-ndhF* spacer (2) and adjacent *ndhF* gene (2). In the *rpoB*,* rpoC2*,* rbcL*,* rps11*,* rpl22*,* ycf1* and *ccsA* genes, a total of ten SNPs cause differences in the amino acid sequence between the CNE and IB *A. montana* samples, while a further five SNPs caused amino acid changes in single samples. Based on an amino acid similarity network^70^, the coding sequence changes between IB and CNE plastomes (mean node distance: 4.1) cause greater change in amino acid function than those not associated with the split (mean node distance: 3.6; Supplementary Table S2.5).

### Variation in *Arnica montana* read datasets

**Fig. 5:**
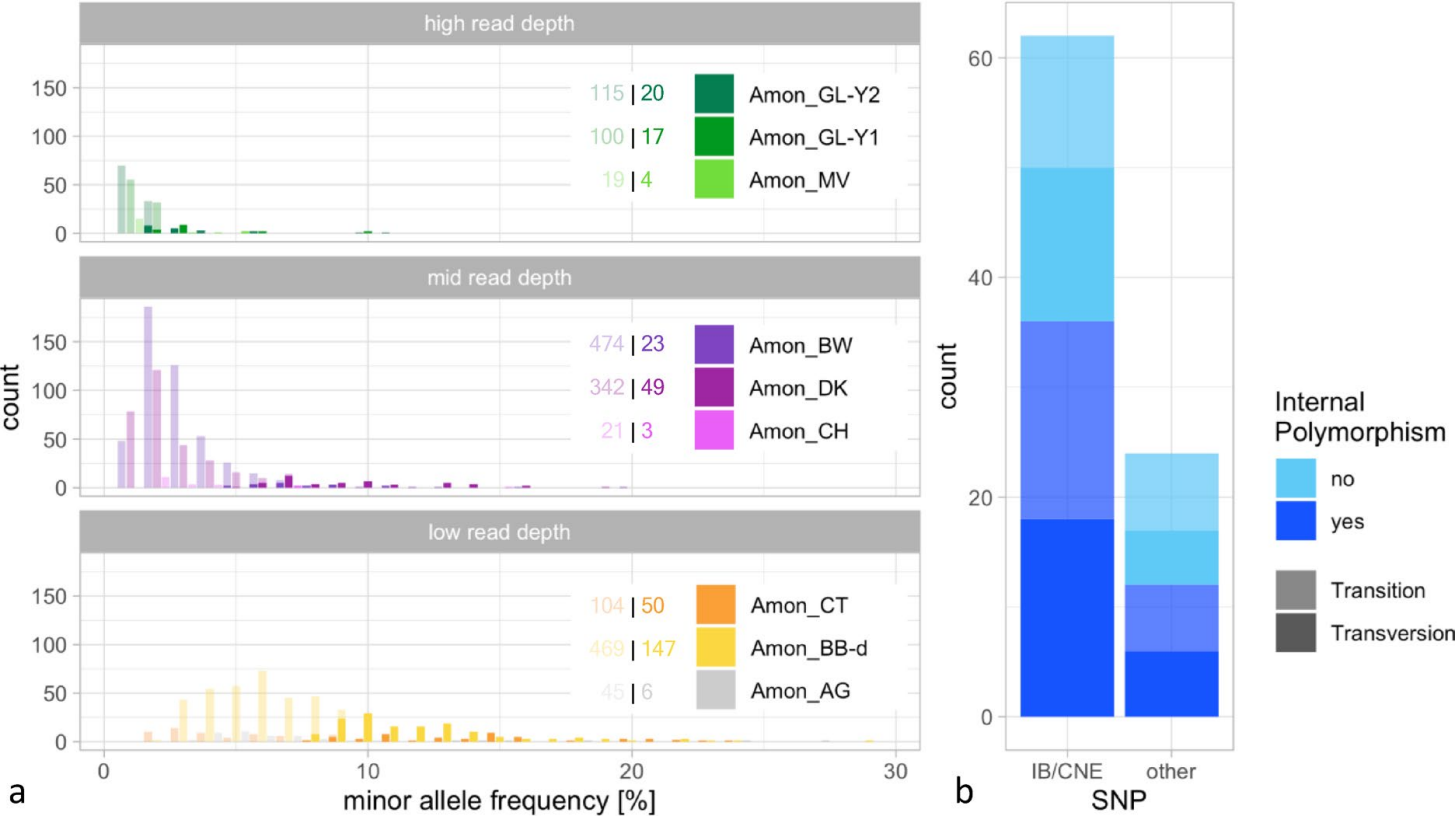
(**a**) Histograms of minor allele frequencies (≥ 0.01) at internally polymorphic SNP sites for all studied *A. montana* plastomes. Samples grouped by plastome mean read depth: High read depth > 1000, mid read depth > 180, low read depth > 65. Solid bars highlight statistically highly significant SNPs, with total numbers of displayed and highly significant SNPs given next to legend. (**b**) Internal polymorphism at sites of *A. montana* within-species SNPs (AM alignment). Loci categorized as showing the IB/CNE split or not (left / right), by presence/absence of internal polymorphism (dark blue / light blue) and by type of SNP (transversion: solid, transition: faded).

We found low-frequency (≥ 1%) variant reads in all *A. montana* read datasets we analyzed. All these internal polymorphisms were located in the single copy regions, with the notable exception of one coding SNP in the 5’-part of the *ycf1* gene, which overlaps into the inverted repeat. Indels were usually associated with copy number variations in homopolymer stretches or microsatellites; our method cannot detect variation for long indels (> 5 bp) or inversions. While the total number of detected internal polymorphisms did not correlate with either the number of input or plastome-mapping reads, the shape of the frequency distribution of alternate SNP alleles seemed correlated with mean read depth: In the three largest datasets included here (Fig. 5a), loci with alternate reads at 1–2% frequency were most common, this distribution shifting its peak to higher frequencies and spreading out with decreasing mean read depth. The maximum alternate read frequencies were > 10% (Amon_MV: 4.7%) in the high (> 1 000 x), > 15% in the mid (> 180 x) and > 20% in the low (> 65 x) mean read depth category. The minimum alternate read frequency required for variants to be statistically highly significant (*p* < 0.01), i.e. unlikely to come from random sequencing errors, showed a similar pattern: from 2% for the highest, it increased to around 8% (Amon_AG: 12.8%) for the lowest mean read depth datasets. Because of the high technical heterogeneity of our data, we did not filter out non-significant polymorphism.

Comparing internal polymorphism across plastomes, the two technical replicates Amon_GL-Y1/Y2 had similar, though not identical patterns of internal polymorphism, with SNPs with higher minor allele frequencies more likely to be shared (see also Supplementary Figs. S1.6, S1.7). The third-largest dataset Amon_MV, containing an exceptionally high proportion of plastome-mapping reads, had comparatively few internal polymorphisms. In the downsampled dataset Amon_BB-d (and Amon_GL-d, Supplementary Fig. S1.6a), the shape of the frequency distribution reflected the low amount of plastome reads in the data, with many more apparently polymorphic loci detected. The only herbarium specimen-derived dataset, Amon_AG, did not show specific patterns, apart from having the lowest mean read depth to begin with. For all samples except Amon_BB-d and Amon_CT, three to seven out of the SNP loci with the highest minor allele frequencies were located at or near a local read depth peak in the *atpI-atpH* spacer (≈ 25 600–25 900 bp from the *psbA* end of the LSC, Supplementary Fig. S1.7b). For the two plastomes with the lowest genetic distance, Amon_DK data contained the Amon_MV variant of both divergent sites at low frequency (SNP: 5.9%, poly-A/T: 15.9%).

At more than half of the sites where we found SNPs between *A. montana* samples, we also found internal polymorphism in individual datasets (Fig. 5b). Read depths at these sites were usually above 100 (Amon_AG: > 65) and minor allele frequencies around 1–5% (Amon_BB-d: ≤ 24.5%), corresponding to at least four reads (Supplementary Table S2.4). We found no transition/transversion bias (Fig. 5b), but a striking preference for pre-existing variants: With one exception where a third allele was present, any alternative allele was identical to the sequence of another *A. montana* plastome at the same locus. For SNPs showing the IB/CNE split, also with one exception, only read datasets from one geographic group contained the sequence of the other as a minor allele, with either group monomorphic in equal frequency. This pattern included the previously studied TC/GT double substitution in the *pafII-cemA* spacer, where we found the GT allele at 100% in all IB samples, and at 4.5% in Amon_DK and 10.3% in Amon_BW.

## Discussion

### PlastidPipeline

Moving further towards findable, accessible, interoperable and reusable (FAIR)^71^ plastome data, we introduced the PlastidPipeline as a taxon-independent tool to standardize and harmonize the *de novo* assembly and annotation of complete plastid genomes in a user-friendly “one-stop shop” pipeline, including both metadata annotation and quality checking steps in an easily adaptable and extensible snakemake framework. In contrast to nuclear genomes, where international networks establish reporting standards^72^, and despite individual attempts^73,74^, there currently are no universal conventions for plastid genomes, and previously published (annotated) sequences should not be taken on trust^36^. To ease reuse and comparison, we propose LSC > IRA > SSC > IRB, with *rbcL* and *ndhF* in forward direction and the inverted repeats named in reading order, as a universal standard for submission to public databases, being aware that this is an artificial structure not reflective of the natural diversity of plastid genome conformations^28,75^. Though it can be adapted to fit individual projects’ needs, this is also the standard output conformation of our pipeline.

Beyond facilitating and standardizing the production of annotated plastid genomes, our pipeline reports statistic and backmapping data, thus easing quality control. From the comparison across our heterogeneous example dataset, 2.2 to 27.5 million 150 bp WGS read pairs should suffice to assemble a plastome ca. 150 000 bp long to a read depth of at least 100, assuming 10% loss of reads in quality filtering, a plastome read content range of [0.02, 0.05] and a minimum: mean read depth ratio of [0.1, 0.5]. For excessively large input files, we recommend truncating them rather than downsampling reads by frequency^36^: Due to the plastid genome’s high copy number per cell, plastome reads tend to be overrepresented in the data to a variable degree (compare Amon_BW and Amon_MV prepared and sequenced in parallel), and would thus get selectively depleted (Table 2). With plastome and similar-sequence nuclear genome reads both present at comparable frequencies, extension-based assembly algorithms, such as employed in GetOrganelle, may be more prone to errors (Fig. 5a). Downsampling also neither sped up assembly: since GetOrganelle will only use as much of the data as needed to produce contigs of the expected size, it may actually stop earlier in a large, but “plastome-biased” whole genome dataset^59^. We recommend also reporting further quality statistics such as plastome read content and minimum read depth, e.g. based on our pipeline’s output.

Potential technical issues with our pipeline are identical to those of the individual tools: GetOrganelle can fail to assemble complete plastomes, sub-optimally assemble homopolymer stretches^32^ or erroneously incorporate nuclear genome reads. This can be remedied by varying the assembly parameters, checking for coverage drops in the backmapping data and, if possible, re-sequencing doubtful sequence stretches with a different method. Using web-server based GeSeq causes a potentially instable dependence on third-party infrastructure, while the tool itself may produce sub-optimal annotations^62^, including a persistently wrong-sense IR annotation and remnant duplicate features as multiple annotation approaches are used, or miss features which are too dissimilar from the curated references. Though our pipeline automatically catches many of these annotation issues, we recommend always manually checking its output: For the two apparently missing tRNA genes, the absence of *trnR-*CCG seems plausible^76^, while the *trnE* gene is essential to plastid functioning^77^ and, by comparison with homologous sequences from other Heliantheae^78^ and *Arabidopsis thaliana*, may indeed be present and functional in all studied samples. As no tool is perfect, we strongly encourage all future users of the PlastidPipeline to make any improvements to the current software which they see fit, while keeping backward compatibility and attribution.

### Evolutionary history of *Arnica montana*

In this study, we report the first fully assembled and annotated chloroplast genomes for eight samples of the European endemic *Arnica montana* (Fig. 3c), as well as for four specimens identified as other *Arnica* species (Fig. 2), and (re-)assemblies for three non-*Arnica* species (Table 1). Phylogenetic analyses of these data support monophyly of *A. montana*, as well as the early branching of northeast Asian *A. unalaschcensis* within the genus (Fig. 3b, Supplementary Fig. S1.4)^41^. The sparsity of the currently available data does not allow further phylogenetic interpretation, e.g. with respect to an origin of the genus *Arnica* in arctic or subarctic western North America^79^, temperate western North America^80^ or the boreal northwest Pacific Rim^41^, or regarding the geographic origin and early diversification of *A. montana*. A comprehensive phylogenetic study will require broader taxonomic and geographic sampling, particularly for wide-spread species such as *A. angustifolia*, as well as complementary nuclear genome evidence^81,82^. In this context, two *A. montana* nuclear reference genomes currently being sequenced will be of great value.

Within *A. montana*, we found evidence for two separate geographic groups, IB and CNE, which show some further internal structure, yet do not coincide with the previously described subspecies^42,55–57^. For IB, the genetic distance between the two sampled plastomes is greater than expected from a comparison with the CNE group, which recalls the observed chemotypic and genetic distinctiveness of populations in Galicia and northern Portugal, even though the putatively distinguishing plastid markers were identical for both our samples. For CNE, genetic distances between plastomes were generally small and gradually declined towards the North (Fig. 4b–d), consistent with northward range expansion as previously suggested based on nuclear microsatellite genetic diversity^44,58^. The observed divergence between IB and CNE plastomes, together with tentative divergence-time estimates (Fig. 3d, Supplementary Fig. S1.5), is consistent with a history of long-term geographic isolation. However, the limited sampling currently available, particularly in Iberia and the area between IB and CNE, does not allow robust inferences regarding refugial locations or demographic history. Additional sampling, especially from southern France and the Pyrenees, together with nuclear genomic data, will be required to evaluate these hypotheses. The few parsimony informative characters and apparent homoplasy we observed in the plastome, as well as low dispersal / strong genetic isolation and, at least periodically, a high rate of clonal reproduction causing increased genetic drift^83^, will remain methodological challenges for phylogeographic studies in *A. montana*. Still, the range-wide evolutionary history of *A. montana*, including its response to past climate change, will be an important guide to its conservation and can only be uncovered with genetic data.

Dating the diversification of *Arnica* remains challenging due to the lack of reliable calibration points. Sedimentary ancient DNA (*sed*aDNA) currently represents the only (sub-)fossil record of the genus, and confirms the presence of *Arnica *sp. in central Alaska since at least 15 000 years^84^, on Svalbard at least 11 000 years ago^85^ and in the Austrian Alps for at least 3 000 years^86^. However, it does not allow further retrospection in time or the discrimination of *Arnica* species. As we used time range estimates^65,69^ for the split between crown-group^41^*A. montana* and *A. chamissonis* as secondary calibration of the split between early-branching *A. unalaschcensis* and all its congeners, all times in our dated phylogeny are systematic underestimates. More comprehensive taxon sampling may remove this latter issue, but robust dating will ultimately require stronger calibration evidence.

### Pan-plastome phylogeography

We demonstrated that using whole plastid genomes for phylogeographic studies is both feasible and advisable, particularly in species with low plastome diversity such as *A. montana*. Choosing only part of a genome to represent the evolution of the whole is not trivial, as, beyond technical constraints and potential homoplasy, the marker regions need to be variable at the right diversity level. Despite the wide spread of polymorphism in the plastomes’ single-copy parts (Fig. 3c), we found five regions with SNPs potentially identifying *A. montana* among congeners, and also distinguishing the IB/CNE geographic groups (Supplementary Table S2.2). Except for the *rbcL* gene, none had previously been proposed as a marker or barcoding region^2,4^, or, to our knowledge, been sequenced in *Arnica*. Most informative regions were coding sequences containing only one or two SNPs, and often showed additional read polymorphism at their intra-specific, even amino-acid-changing, SNPs. Given this low polymorphism and high potential for artefacts in marker-based approaches, we recommend combining whole plastomes with nuclear genomic evidence.

Read polymorphism within samples is an as-yet mostly unexplored aspect of plastome diversity. Potential heteroplasmy only recently started to gain attention^22–25^, yet given its importance for understanding the evolution of widely-used genetic markers, its potential to explain apparent missense mutations, such as the premature *matK* stop codon we observed in Amon_CH and Gtul_MX, and following the increasing number of pan-plastome studies analyzing differences between closely related samples^16–19^, we expect more data to become available in the future [5]. So far, distinguishing true heteroplasmy from technical artefacts such as read error, sequence-dependent polymerase bias or mismapping remains difficult: though we found slightly increased local read depths at SNPs with the highest minor allele frequencies, the pervasiveness and highly significant minor allele frequencies of internally polymorphic SNPs, their numerical independence from the overall number of reads and re-occurrence at homologous loci across our widely different datasets, and the apparent directionality of changes associated with the IB/CNE split, all speak for a biological cause. In contrast to our expectation, minor allele frequency spectra did not show multiple (error vs. signal) peaks and were strongly dependent on the mean read depth of the dataset, so that simple frequency cutoffs across heterogeneous read datasets^23–25^, even from the same experiment (e.g. Amon_BW and Amon_MV, Fig. 5a), could be misleading. Further methodological development may be necessary to turn anecdotical observations such as ours into complementary evidence in phylogeographic studies. In the meantime, we suggest routinely checking for read polymorphism in backmapping data of plastome assemblies, such as provided by the PlastidPipeline.

## Methods

### Sequence data

The raw sequence data we used for this study was either acquired in our own labs, i.e. three samples of *A. montana* from Germany and one from NW Spain, obtained directly from the authors (PhyloAlps) or downloaded from GenBank or the European Nucleotide Archive (ENA). The geographical origin of *Arnica* samples relative to each species’ distribution area is displayed in Fig. 2, with more details given in Table 1. This data includes all currently (March 2025) publicly available WGS datasets for the genus *Arnica*.

Total genomic DNA came either from herbarium vouchers (PhyloNorway, Gtul_MX, Vcar_CA), flash-frozen material intended for nuclear genome sequencing (samples Amon_BB and Amon_GL) or silica-dried leaf samples (all others). Library preparation for Illumina sequencing usually involved additional PCR amplification of the sample (e.g. Illumina TruSeq Nano DNA Kit for Amon_BW and Amon_MV), except for the two nuclear genome samples where the Illumina TruSeq PCR-free kit was used. The German samples (Amon_BB, Amon_MV, Amon_BW) were sequenced on an Illumina HiSeq X at Macrogen Inc., Seoul, whereas the NW Spanish specimen (Amon_GL) was sequenced on an Illumina NovaSeq 6000 at the Spanish National Center of Genomic Analysis (Centro Nacional de Análisis Genómico, CNAG), Barcelona. Technical information about each read dataset, including read lengths (100 bp for PhyloNorway, 150 bp for all others) and read pair numbers, is given in Table 2.

Because of the size of the nuclear genome datasets (Amon_BB, Amon_GL), we also tested bioinformatically downsampled versions of the full data (Amon_BB-d, Amon_GL-d) obtained with bbnorm^87^ and a target read depth of 200x (following a previous study^36^). For Amon_GL, data was available from multiple sequencing runs of the same sample, which we treat as technical replicates and distinguish by the last character of the run + lane identifiers, i.e. Amon_GL-71 to -74 for HFYM3DSX7_1 to _4 and Amon_GL-Y1 and -Y2 for HGM7JDMXY_1 and _2. The Amon_GL dataset was concatenated from part of lanes one and three of HFYM3DSX7.

### PlastidPipeline

We developed the PlastidPipeline to create a cohesive pipeline for the reproducible *de novo* assembly and annotation of plastid genomes. It uses snakemake^61^, a Python based workflow management system that allows for independent cross validation, adaptation and automation of data analysis. Due to their modular nature, snakemake pipelines easily allow input at different steps, or to resume the analysis with changed parameters starting from an intermediate result. Parameter settings for each step are fully configurable by the user through a configuration file, defaulting to the standard settings of the respective tool unless specified otherwise. For functionality not covered by the available software tools, e.g. structural standardization of the plastome sequence, the pipeline contains additional bash or Python scripts written by the authors, particularly using Biopython and Selenium^88–90^. The PlastidPipeline is available from GitHub (https://github.com/sannalda/plastidpipeline) and was developed and tested on FU Berlin’s Curta HPC system^91^.

A schematic overview of the PlastidPipeline is given in Fig. 1. Starting from the most common input, i.e. raw paired-end sequence reads in .fastq(.gz) file format, it typically performs the following steps: first, reads are trimmed and quality filtered using fastp^92^. Second, GetOrganelle^59^ is used to assemble the plastid genome: briefly, reads are first mapped to standard seed sequences from an embryophyte plastome database. Through iterative extension with SPAdes^93^, more target-associated reads are added and contigs assembled *de novo*. Non-target contigs are filtered out and the remaining targeted organelle contigs are enchained into a complete circular plastid genome. Critical GetOrganelle parameters for the assembly are the number of extension rounds -R, which we doubled to 30 by default in the PlastidPipeline, and the “word size” -w, i.e. the length of sequence used to detect read similarity, which can be set for each pipeline run. Especially if the assembly fails, the contig graphs can be manually checked with Bandage^94^ (here 0.9.0) and -R increased / -w decreased against fragmentation, or the inverse against entanglement.

The third step in the PlastidPipeline is to standardize the orientation of the two single copy regions and to annotate the plastome sequence: first, GeSeq^60^ is used for a preliminary annotation, which identifies the four parts of the plastid genome and one gene each in the LSC and SSC, specified by the user along with their intended orientation. By default, these are the highly conserved chloroplast household genes *rbcL* in the LSC and *ndhF* in the SSC, both in forward orientation (i.e. haplotype A^28^). According to the linearized isomorph randomly produced by GetOrganelle (four possibilities, see Supplementary Fig. S1.1–3), the plastome parts are split up, potentially reversed and reassembled into the required form. Subsequently, GeSeq is called again to annotate the standardized plastome sequence, and a script is run to test for the completeness / duplication of annotations (“blatX/N” annotations being kept preferentially by default), verify the plausibility of coding sequence features and to correct potential mislabeling of the IRs. In a test on one sequence, GeSeq produced a more complete annotation of our data compared to its most popular command line alternative, PGA^95^, though it is currently only available as a web application and thus cannot be directly integrated into an automated software pipeline. The PlastidPipeline therefore contains a script which simulates using the GeSeq web interface, setting parameters (e.g. including both “blat” and “Chloe” annotators) and uploading/downloading files according to the user’s specifications. It also automatically corrects names and orientation of the IRs, removes duplicate annotations and produces a test report for the presence/absence of standard plastid genes^74^, start/stop codons, reading frame lengths and translations of coding sequences.

The fourth step of the PlastidPipeline outputs backmapping information for the input reads, using Bowtie 2 and samtools^96,97^. This is intended for further quality checks, e.g. the detection of apparent heteroplasmy or regions with low or uneven support, for which a figure and a per-base read count file are produced. Finally, the fifth step of the pipeline optionally adds sample metadata, as specified in an additional input text file per sample, to the header of the final annotated .gb (Genbank) file the PlastidPipeline produces per input dataset.

Though the produced files are directly ready for use in further analyses (e.g. whole plastome alignment, phylogenies), we strongly recommend to first visually inspect them for any undetected errors. To submit the final plastid genomes to an INSDC database, the output Genbank files still have to be converted according to the respective database’s requirements. Sequence submission is therefore not included in the PlastidPipeline. To format our data for submission to ENA, we used custom scripts including file conversion with Biopython^89^ and the ENA webin-cli programmatic submission interface.

### Data analysis

We visually inspected the plastid genomes produced by the PlastidPipeline in Geneious Prime 2025.0.3, using its MAFFT^98,99^ interface to create preliminary alignments. The assembled sequences for Vcar_CA and Gtul_MX we compared with previous NOVOPlasty assemblies^66^. For detailed analyses, we then built three separate alignments at different levels: excluding the technical replicates, we first aligned only the *A. montana* sequences (“*Arnica montana*” = AM alignment), then, keeping previous gaps, successively added first the other *Arnica* plastomes (“all *Arnica*” = AA alignment), and finally the three non-*Arnica* samples (“all samples” = AS alignment). At each step, we manually checked the alignment for undetected short indels or inversions (> 2 bp long), harmonized gap limits and removed the second IR (IRB) while, in AS, keeping variable sites at the beginning and end of the IRB to account for IR length variation in the non-*Arnica* outgroup. For the AM alignment which comprised the most similar sequences, we kept all polymorphic characters including gaps. We encoded indels/inversions as unordered multistate characters (MSI)^100^, to account in the best possible way also for complex inversions and sequentially overlapping gaps. For the AA alignment, we only kept gaps part of two-state characters, and removed all multistate characters involving gap “alleles”, i.e. mostly length variable A/T-homopolymers, from the analysis. For the AS alignment, we removed all characters with gaps in at least one sample, keeping only unambiguously aligned single nucleotide polymorphisms (SNPs). For all alignments, we calculated Pairwise Character Differences (PCD = “Hamming distance”) in Geneious, excluding and, where applicable, including indels/inversions, with gaps as a fifth character state. This distance measure gives equal weight to transition, transversion and indel characters. For comparison (not used in evolutionary inferences), we mapped all publicly available sequences of *A. montana* plastome marker regions (GenBank/ENA accessions^41,42,45,101^: Supplementary Table S2.8) to our full sequence alignment. One previous plastome assembly for Amon_DK, marked as unverified (MT899992), was incomplete and also not used in our analyses.

Based on the AS and AA alignments, we employed different criteria to infer phylogenetic trees, using the Geneious plugins for:

- PAUP* 4.0a168^102^ for Neighbor Joining and Parsimony, performing a heuristic search from a starting tree obtained by stepwise addition of the furthest sample,
- MrBayes 3.2.6^103^ for Bayesian Posterior Probability, running four heated Markov chains with 0.2 temperature for 1 100 000 steps with a 100 000 step burn-in,
- PhyML 3.3.20180621^104^ for Maximum Likelihood with chi^2^ branch stability and
- RAxML 8.2.11^105^ for Maximum Likelihood with different data partitioning schemes (unpartitioned, AS: coding/non-coding, AA: LSC/IR/SSC).

All other settings were kept as similar as possible across all methods, i.e. using the general time reversible model (GTR, also suggested by modeltesting^106^) with no invariable sites and, where available, gamma estimation, a random starting tree and, except for MrBayes and PhyML, bootstrapping with 10 000 replicates to obtain branch support values. As the partitioning schemes for RAxML produced roughly the same result (topology, bootstrap values) as the unpartitioned scheme, we retained the latter. For the AS alignment, the three non-*Arnica* samples were set as the (monophyletic) outgroup. Since the *A. unalaschcensis* (Auna_AK) plastome consistently branched first in the *Arnica* clade in all AS-based trees (Supplementary Fig. S1.4), this plastome served as outgroup for our inference of the *Arnica* phylogenetic tree based on the AA alignment. To date a posterior probability-based phylogeny of all *Arnica* samples, we used the same general settings and BEAST 2.7.7. and associated tools (BEAUti, Tracer, TreeAnnotator) with both a strict and a relaxed molecular clock model^107,108^, visualizing the result with FigTree^109^: since it is currently the only data available for (secondary) calibration, we used the range of estimates for the split between the *A. montana / A. chamissonis* transcriptomes given in recent Asteraceae phylotranscriptomics studies^65,69^, i.e. [3.83, 9.54] Ma, or [7.73, 11.92] Ma for a dataset with increased species sampling, as uniform prior for the divergence between the stem group, represented by *A. unalaschcensis*, and the other *Arnica* crown group species. We further inferred a parsimony network^110^ among *A. montana* plastomes with the R package haplotypes 1.1.3.1 on the AM-MSI alignment.

Based on the backmapping .bam files produced by the PlastidPipeline, we detected sequence variants with minor allele frequencies ≥ 0.01 for the *A. montana* plastomes with Geneious. We focused our analysis on SNPs, excluding the first 100 bases of the LSC, where mapping to the start of the linearized sequence is incomplete, and duplicate information from the IRB. As the largest dataset (Amon_BB) could not be analysed in this way due to its size, we used the Amon_BB-d dataset to represent Amon_BB, and both Amon_GL-Y1 and Amon_GL-Y2 to represent Amon_GL. Supplementary Figs. S1.6, S1.7 and Supplementary Table S2.7 show a comparison of intra-individual SNPs detected with all Amon_GL datasets. To test the statistical significance of the detected variants, we calculated their probability (p-value) to originate from coincident read errors assuming these are binomially distributed, and compared it to a significance level (acceptable false-positive error rate) of 0.01, divided by the maximum LSC+SSC+IR length of all *A. montana* plastomes in this study (Bonferroni correction for multiple testing). We calculated the p-values using the base R function dbinom, with the number of observations equal to the count of variant reads, the number of trials equal to the total read depth at the variant base, and the probability of success equal to the base-calling error corresponding to one third (as there are three alternative bases) of the base-calling error probability associated with the minimum Phred quality score used in the fastp read quality filtering step. An R script to replicate our statistical test is included as Supplementary File S4.

To run statistical analyses and draw diagrams, we used R 4.4.2 with ggplot2^111,112^, UpSet (for Supplementary Fig. S1.7a)^113^ and the geosphere 1.5–18 and vegan 2.6–6.1 packages for the Mantel plot and tests^114,115^. The pan-plastome map in Fig. 3c was modified from an OGDraw plot^116^. For geographic maps, we loaded and filtered observation data from gbif^117^ with the rgbif 3.8.0 and CoordinateCleaner 3.0.1 R packages^118,119^, combining it in QGIS 3.36 with background map data from www.naturalearth.org^120^.

## Supplementary Information

Below is the link to the electronic supplementary material.

Supplementary Material 1

Supplementary Material 2

Supplementary Material 3

Supplementary Material 4

## Data Availability

All data included in this study are available from the INSDC databases under the study reference number PRJEB89801 and the reference numbers provided in the text, in Table 2 and in Supplementary Table S2.1. The PlastidPipeline and its documentation are available on Github, https://github.com/sannalda/plastidpipeline, and as a first release on Zenodo, https://doi.org/10.5281/zenodo.21672765.
