# Supplementary material for: Towards whole plastome phylogeography: resolving small genetic distances among European Arnica montana L. with the PlastidPipeline
Source: Sci Rep. 2026 Aug 15;16:25523. doi: 10.1038/s41598-026-65517-1 (PMC13477478; doi:10.1038/s41598-026-65517-1)
Supplement: Supplementary file 1 [file 41598_2026_65517_MOESM1_ESM.pdf]

Supplementary Material S1

Chloroplast structural variants

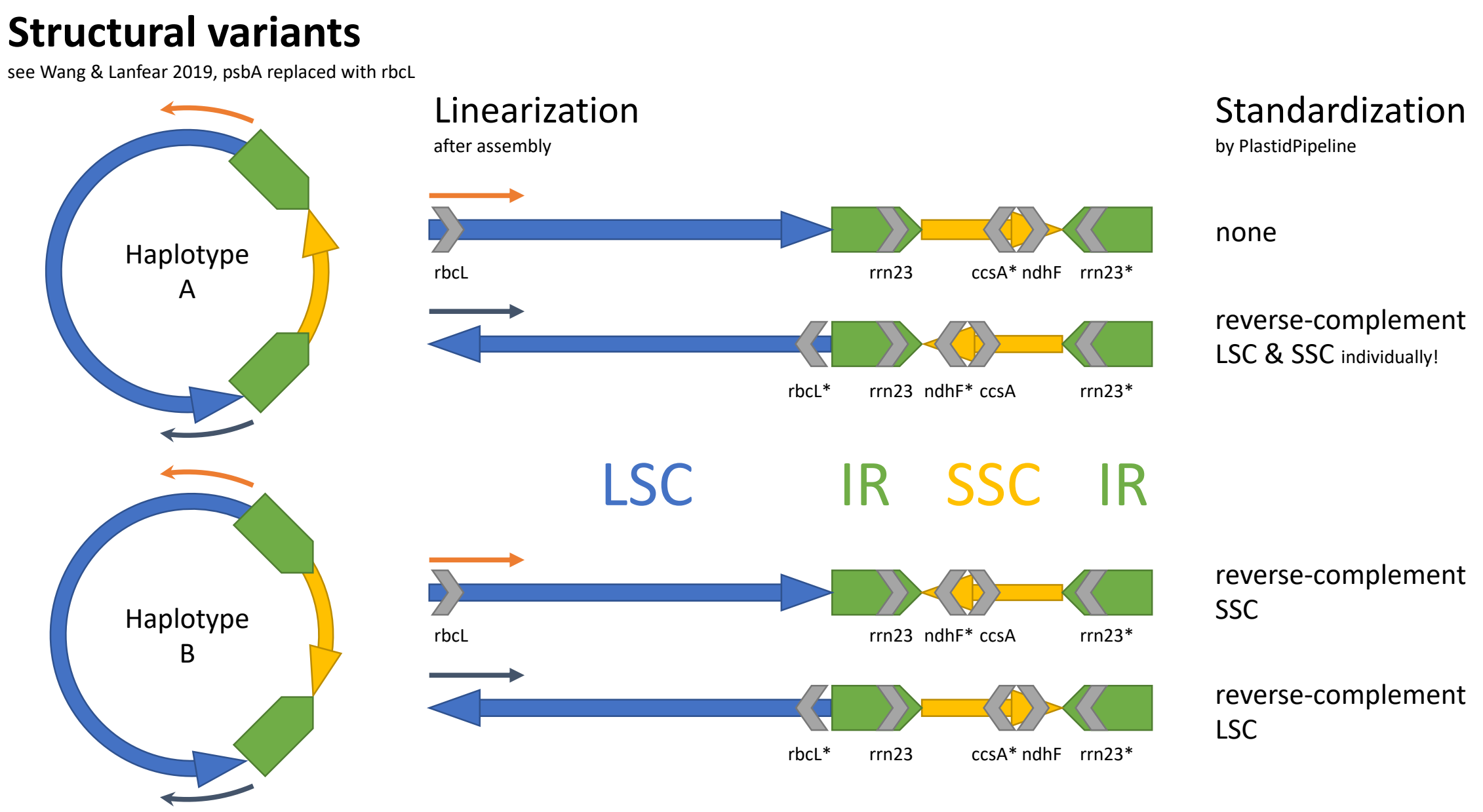

Figure S1.1 Schematic overview of structural variants, their linearizations and the respective structural standardization operations performed by the PlastidPipeline. Blue: Long Single Copy (LSC) region – yellow: Short Single Copy (SSC) region – green: Inverted Repeat (IR) regions – grey: genes. Orange and dark blue arrows: Possible directions for linearization of the circular plastome into the format LSC-IR-SSC-IR, e.g. as output by GetOrganelle. Note that all four linear conformations correspond to naturally occurring plastome structures.

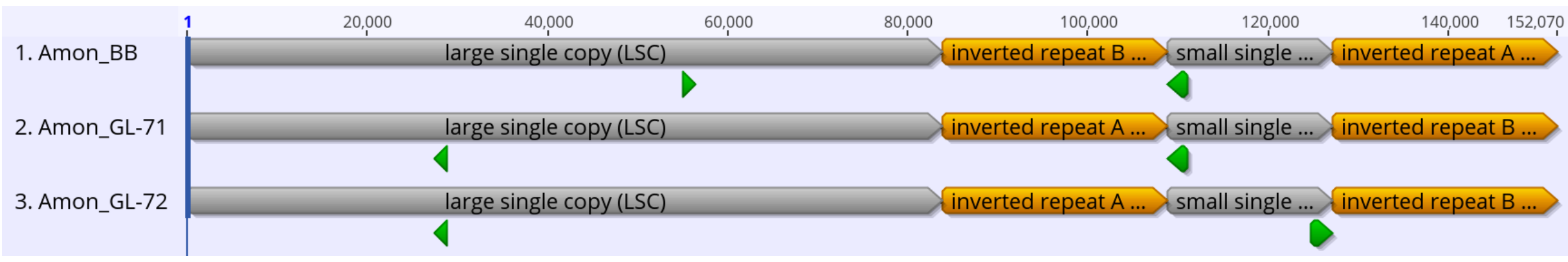

Figure S1.2 Examples for alternative linear plastome conformations before structural standardization (Geneious screenshot). Raw output from GetOrganelle with preliminary GeSeq annotation, filtered for structural features (grey – LSC, SSC, orange – IRA, IRB) and the two genes (green – *rbcL, ndhA*) used by default for standardization in the PlastidPipeline.

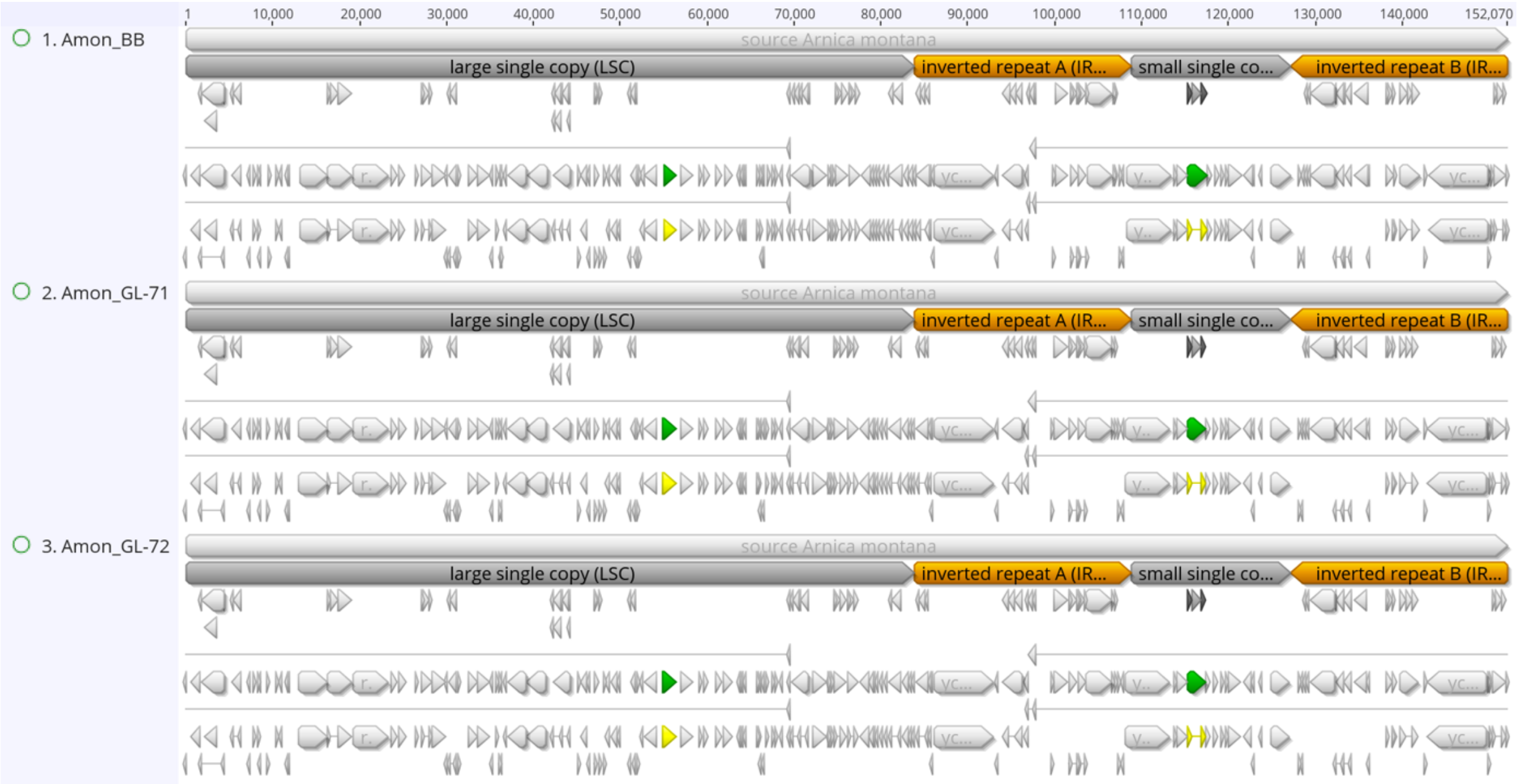

Figure S1.3 Examples of automatically standardized, fully annotated plastomes output by the PlastidPipeline (Geneious screenshot). Structural annotations and standardization genes (incl. CDS, exons, intron) highlighted.

Additional phylogenetic trees

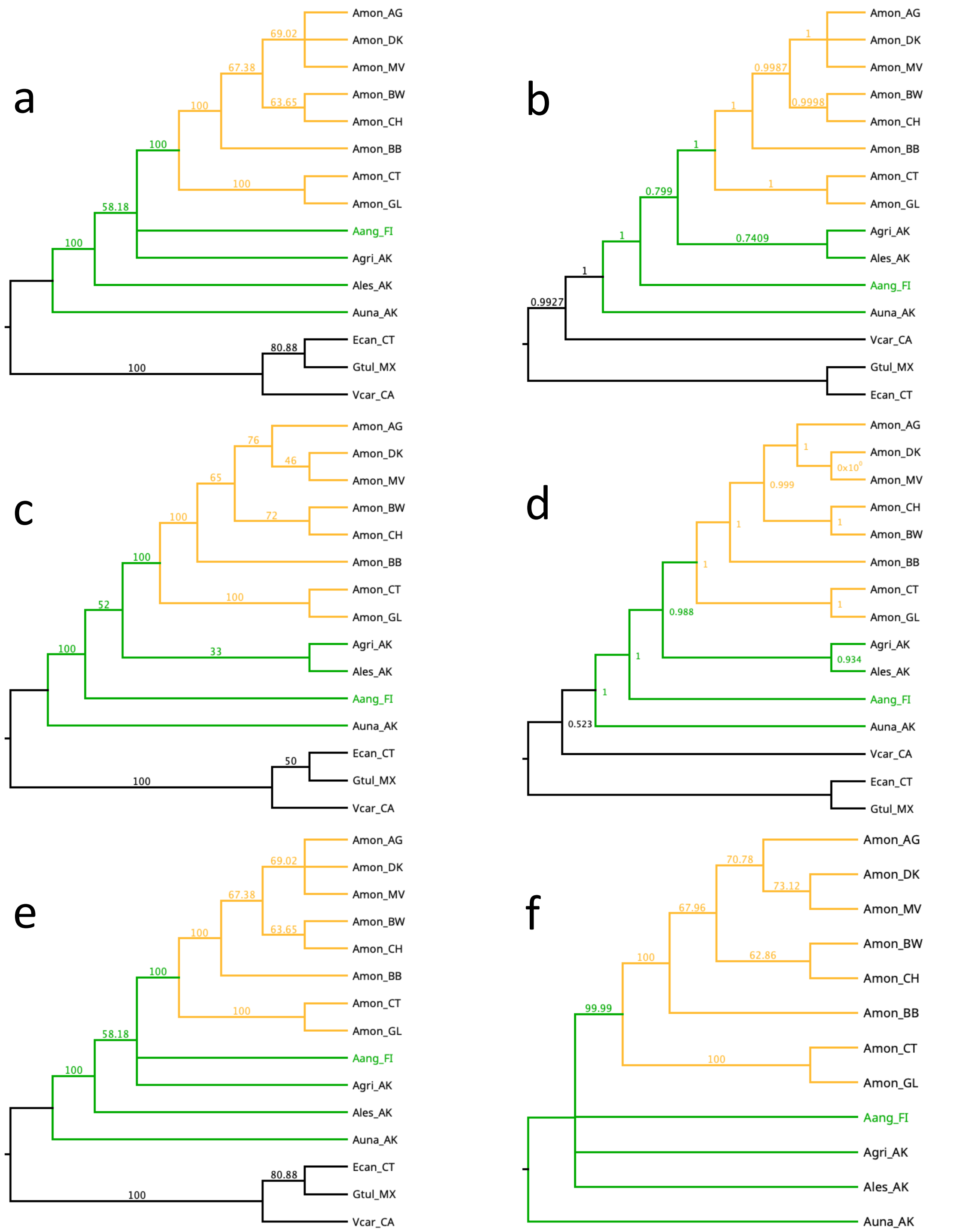

Figure S1.4 Additional phylogenetic trees based on all samples (AS alignment with non-*Arnica* species as outgroup, based on SNPs, a-e) or all genus *Arnica* samples (AA alignment with *A. unalaschcensis* as outgroup, based on SNPs + 2-state indels, f). *A. angustifolia* sample highlighted for comparison. a. PAUP* Parsimony tree with bootstrap support values (10 000 replicates). b. MrBayes tree with posterior probabilities. c. RAxML Maximum Likelihood tree with bootstrap values (10 000 replicates). d. PhyML Maximum Likelihood tree with Chi2-based branch support values. e. PAUP* Neighbor-Joining tree with bootstrap support values (10 000 replicates, support < 50% shown as polytomy). f. PAUP* Neighbor-Joining tree with bootstrap support values (10 000 replicates, support < 50% shown as polytomy – compare with Fig. 2b in the main text inferred from the same data).

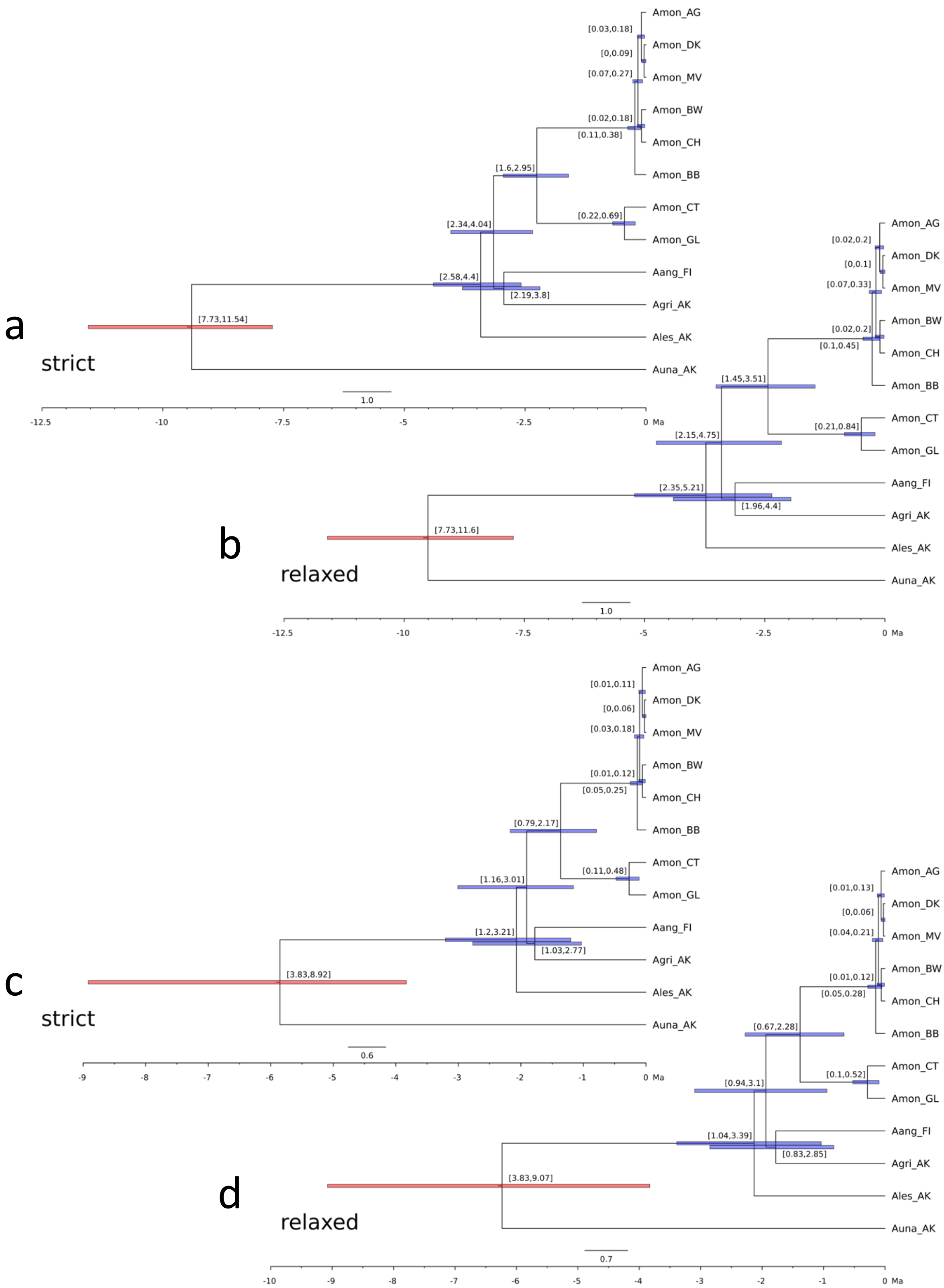

Figure S1.5 Time-calibrated phylogeny, based on two alternative calibrations, assuming a strict (a,c) or a relaxed (b,d) molecular clock. BEAST 2.7.7. analysis of the unpartitioned AA alignment, using the extremes of the date suggestions for the *A. montana / A. chamissonis* split in (a,b) Zhang et al. 2024 ([7.73, 11.92] Ma) or (c,d) Zhang et al. 2021 ([3.83, 9.54] Ma) as uniform prior range for the secondary calibration of the root of the tree (red age bar). Node age bars: age range, node age intervals: 95% confidence interval. Fig. 2d in the main text is based on Figures S1.5b and S1.5d.

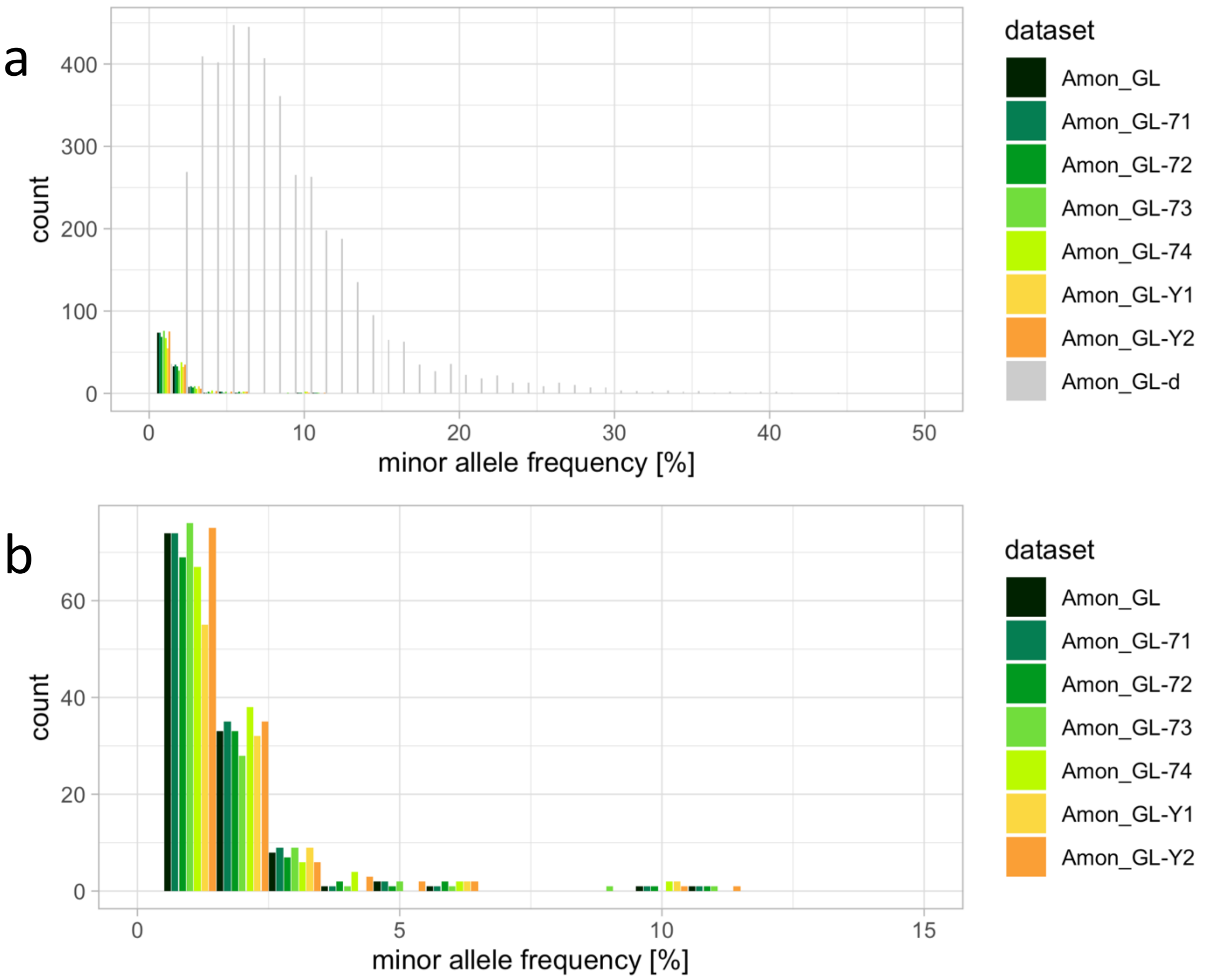

Figure S1.6. Histograms of minor allele frequencies at intra-individual SNP sites for all available read datasets for the Amon_GL *A. montana* plastome. (a) overview including the downsampled dataset, Amon_GL-d, and (b) zoom into the aggregated (Amon_GL) and original (all others) datasets from the same physical sample. Mean plastome read depth ranges from 385.3 for Amon_GL-d, across > 3 000 for Amon_GL-Y1/2 and > 10 000 for Amon_GL-71 to 74, to 19 637.9 for Amon_GL.

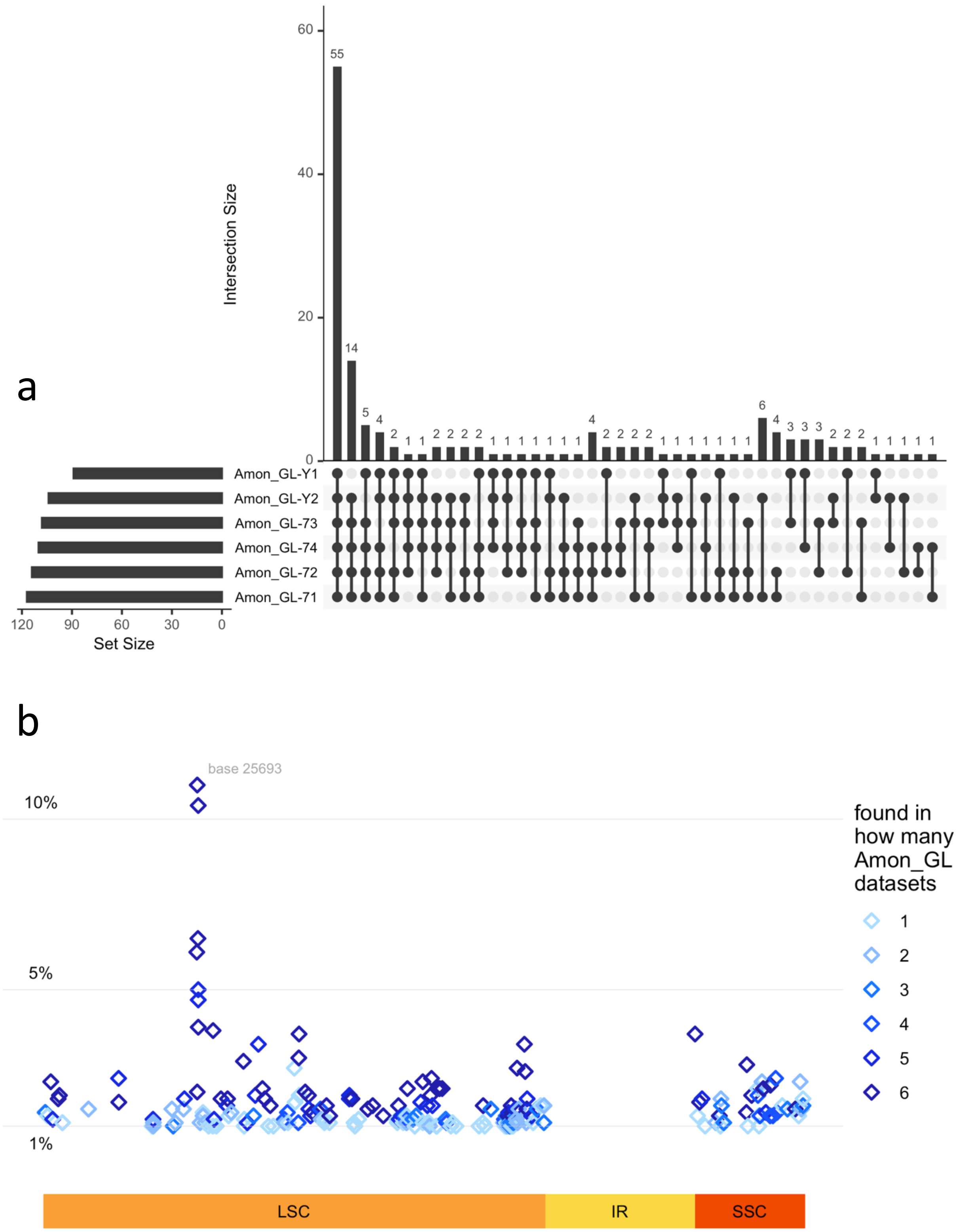

Figure S1.7. Comparison between intra-individual SNPs found within technical replicate read datasets for Amon_GL. Includes only original sequencing datasets for the same physical sample, aggregate and downsampled datasets excluded. (a) Set diagram of SNPs shared between original read datasets, and (b) SNP position (x-axis), maximum minor allele frequency across datasets (y-axis) and number of datasets where detected (color, darker=found in more datasets). The highest minor allele frequencies (>10% in all datasets) were found in the *atpI-atpH* spacer.
